# Dual-Photosensitizer Antimicrobial Photodynamic Therapy (DaPDT) and Its Combination with Antibiotics: A New Investigation Modality Against *Klebsiella pneumoniae*

**DOI:** 10.3390/pharmaceutics18050587

**Published:** 2026-05-09

**Authors:** Koteswara Rao Yerra, Vanderlei S. Bagnato

**Affiliations:** 1 Department of Biomedical Engineering, College of Engineering, Texas A&M University, College Station, TX 77843, USA; 2 São Carlos Institute of Physics, University of São Paulo, São Carlos 13566-590, SP, Brazil

**Keywords:** *Klebsiella pneumoniae*, antimicrobial photodynamic therapy (aPDT), combining two photosensitizers (PSs) with antibiotics

## Abstract

**Background/Objectives**: *Klebsiella pneumoniae* is a major pathogen involved in both acute and chronic infections, characterized by high incidence and significant clinical severity. Over the past decade, resistance to traditional antimicrobial treatments has risen rapidly, highlighting the urgent need for innovative approaches. Light-based antimicrobial strategies, including antimicrobial photodynamic therapy (aPDT), offer a promising approach for addressing drug-resistant bacteria. Combining two photosensitizers (PSs) with antibiotics synergistically enhances ROS generation and multi-target bacterial damage, achieving superior antimicrobial efficacy at reduced PS, light and antibiotic doses while limiting resistance development. We evaluated the efficacy of aPDT using the photosensitizers (PSs) methylene blue (MB) and Photodithazine (PDZ), either alone or in combination with the antibiotic ciprofloxacin (CIP), gentamicin (GEN), or ceftriaxone (CEF), against *K. pneumoniae*. **Methods**: Bacterial suspensions were treated with PDZ (25–200 µg/mL) and/or MB (5–20 µg/mL) in the presence of CIP (0.005–4 µg/mL), GEN (0.5–16 µg/mL), or CEF (0.5–16 µg/mL), followed by irradiation at either 15 J/cm^2^ or 30 J/cm^2^. Bacterial survival was assessed by colony-forming unit (CFU/mL) quantification. **Results**: The combined application of photosensitizers and antibiotics demonstrated a synergistic bactericidal effect against planktonic *K. pneumoniae*. The combined use of two PSs with antibiotics markedly reduced the antibiotic dose required to achieve a comparable bactericidal effect. **Conclusions**: This study highlights the potential of combining aPDT with conventional antibiotics as a promising strategy to combat drug-resistant infections, offering enhanced antimicrobial efficacy while allowing for reduced antibiotic dosages to achieve comparable therapeutic outcomes.

## 1. Introduction

Bacterial infections pose an increasing global health threat, primarily due to the rapid rise and spread of antimicrobial resistance (AMR). This escalating crisis undermines the foundation of modern healthcare, resulting in higher rates of illness and mortality and economic pressure on health systems worldwide [1]. The widespread misuse and overuse of antibiotics in medicine, agriculture, and environmental management have heightened selective pressures that promote the development of drug-resistant microorganisms [2]. Simultaneously, bacterial reproduction, natural mutations, and horizontal gene transfer under antibiotic stress have accelerated the emergence of multidrug-resistant (MDR) pathogens, including so-called “superbugs” [3]. As a result, treatment failures are becoming more frequent with various infectious diseases, such as pneumonia. The World Health Organization warns that many key pathogens now resist multiple classes of antibiotics, limiting treatment options and emphasizing the urgent need for global efforts to combat this problem [4]. While discovering new antimicrobial drugs remains crucial, microbial adaptation is outpacing innovation.

Antimicrobial photodynamic therapy (aPDT) has emerged as a promising nontraditional therapeutic strategy with potent activity against a broad spectrum of infectious organisms [5]. aPDT relies on the light activation of an exogenous photosensitizer (PS), which, upon excitation, generates cytotoxic species through type II photochemical reactions that produce singlet oxygen (^1^O_2_), or type I pathways involving electron transfer and additional reactive oxygen species (ROS) [6]. These oxidative processes damage essential biomolecules, lipids, proteins, and nucleic acids, leading to microbial inactivation. Importantly, the photodynamic mechanism of action bypasses conventional antibiotic targets, thereby minimizing the likelihood of cross-resistance and reducing selective pressure for new resistance traits [6]. Extensive experimental and preclinical evidence demonstrates that aPDT effectively eradicates both planktonic bacteria and structured biofilms, including those formed by antibiotic-resistant strains, positioning it as a compelling adjunct or alternative to current antimicrobial therapies [7].

A wide range of PS structures have been investigated for aPDT applications, ranging from classic chromophores such as the phenothiazinium dye methylene blue (MB), to xanthene derivatives like Rose Bengal, to more advanced cationic tetrapyrrolic derivatives including porphyrins, phthalocyanines, and bacteriochlorins. Among the clinically accessible PSs, MB is notable for its strong antimicrobial activity, including efficacy against drug-resistant bacterial phenotypes refractory to conventional treatments [8]. Photodithazine (PDZ), a second-generation chlorine e6-based PS, similarly demonstrates high antimicrobial activity with low dark toxicity [9]. Recent therapeutic strategies increasingly explore dual-PS systems and PS–antibiotic combinations to harness complementary ROS generation, broaden absorption profiles, and systematically engage multiple antimicrobial mechanisms. Indeed, several reports have described additive or synergistic enhancement when combining PSs or pairing aPDT with antibiotics [10,11]. The MB-PDZ combination is particularly attractive due to its favorable biocompatibility, well-characterized photochemical behavior, and overlapping absorption maxima near ~650 nm, enabling efficient co-activation using clinically relevant light sources. Moreover, their action on distinct bacterial targets may synergistically enhance antimicrobial efficacy.

*Klebsiella pneumoniae* represents one of the most challenging MDR pathogens in clinical settings [12]. It is a leading cause of hospital- and community-acquired pneumonia, urinary tract infections, and life-threatening systemic infections. Its capacity to form strong biofilms contributes to antibiotic tolerance and therapeutic failure. Strains of *K. pneumoniae* producing extended-spectrum β-lactamases (ESBLs) and carbapenemases (KPCs) are classified by the WHO as critical priority pathogens, with associated mortality rates reaching 30% and 70% in severe infections [12]. These concerns highlight the urgent need for adjunctive or alternative strategies that can overcome classical antibiotic resistance mechanisms. Although aPDT has demonstrated strong promise against Gram-negative bacteria, single-PS systems face inherent limitations, including oxygen dependence, restricted tissue penetration, and potential reduction under certain microenvironmental conditions [13]. Dual-photosensitizer photodynamic therapy (DaPDT) combined with antibiotics has emerged as a promising strategy to enhance antimicrobial efficacy, particularly against multidrug-resistant (MDR) pathogens [14]. In DaPDT, distinct PSs can localize to different cellular targets such as the membrane, nucleic acids, and proteins, while generating complementary ROS, thereby inducing widespread and multi-site bacterial damage, including disruption of biofilms [14]. This multi-target, non-specific oxidative mechanism reduces the likelihood of stable resistance development, and its combination with antibiotics further limits bacterial adaptive responses. Moreover, DaPDT-mediated increases in membrane permeability and biofilm destabilization facilitate improved antibiotics penetration, enabling effective bacterial killing at reduced antibiotic concentrations [14]. However, the interaction among PSs, as well as that between PSs and antibiotics, remains insufficiently understood, with the literature describing outcomes ranging from synergistic improvement to antagonistic interference.

In this study, we evaluate the in vitro efficacy of a dual-PS aPDT system combining MB and PDZ with three clinically relevant antibiotics—ciprofloxacin (CIP), gentamicin (GEN), and ceftriaxone (CEF)—against *K. pneumoniae*. The MB-PDZ pair was selected based on their well-characterized photodynamic properties, overlapping red region absorption peaks (~650 nm), and demonstrated antimicrobial efficacy in prior studies [15]. Our primary objective is to determine whether combined photodynamic–antibiotic regimens enhance bacterial killing relative to monotherapies and to elucidate their potential as a strategy for combating MDR *K. pneumoniae* by characterizing the interactions of optimized combination therapeutics capable of overcoming antibiotic resistance and strengthening the clinical utility of aPDT.

## 2. Materials and Methods

The experimental methodology was adapted from two previously published studies by our research group and is summarized below.

### 2.1. Chemicals

In our study, we used two photosensitizer (PS) combinations. The photosensitizer methylene blue (MB) was acquired from Sigma-Aldrich (St. Louis, MO, USA) (USP Reference Standard, cat# 1428008). A stock solution of MB (50 µg/mL) was prepared using sterile water and stored at 4 °C in the dark. Photodithazine^®^ (PDZ) (also known as Fotoditazin^®^) was obtained through scientific collaboration with the Optics group of the Sao Carlos Institute of Physics, USP. It was in 5 mg/mL stock solutions and stored at 4 °C. Diluted PDZ was prepared at a concentration of 400 µg/mL in distilled water and subsequently diluted in distilled water to achieve the desired concentrations. Mixtures of the MB and PDZ solutions were obtained through dilution from the stock solutions using PBS as a solvent. Photosensitizer solutions were prepared in microtubes wrapped in aluminum foil to safeguard against light exposure during the experiment. The antibiotics, ciprofloxacin (CIP, cat# A208061) was purchased from AmBeed (Buffalo Grove, IL, USA), gentamicin (GEN, Gentamicin sulfate, cat# 455310010) was purchased from Fisher Scientific (Fair Lawn, NJ, USA), and ceftriaxone (CEF, ceftriaxone disodium salt hemi heptahydrate, cat# 455420010) was purchased from Fisher Scientific (Fair Lawn, NJ, USA). The stock solutions were made from powder dissolved in sterile distilled water and homogenized by vertexing. Stock solutions of antibiotics were freshly prepared each day in distilled water (dH_2_O) at 1 mg/mL. These concentrations were chosen based on our previously published experiments performed in our laboratory. Phosphate-buffered saline (PBS) was used for microbial cell suspensions and serial dilution. All solutions were prepared and handled under light-restricted conditions.

### 2.2. Bacterial Culture

The Gram-negative bacterium *Klebsiella pneumoniae* subsp. *Pneumoniae* was used in this study and was obtained from the American Type Culture Collection (ATCC^®^ 13883™). A single colony of *K. pneumoniae* from a brain heart infusion agar (BHIA) plate was incubated in brain heart infusion broth (BHIB) at 37 °C overnight in a 5% CO_2_ atmosphere on a shaker incubator at 180 rpm (MaxQ 6000, Thermo Fisher Scientific, Waltham, MA, USA). Then the cells were collected and centrifuged at 4000 rpm for 5 min and resuspended in phosphate-buffered saline (PBS). The optical density of the *K. pneumoniae* suspensions was adjusted to 0.2–0.3 at 600 nm (OD 600, Agilent Cary 60 UV-Vis, Agilent Technologies Inc., Santa Clara, CA, USA), corresponding to approximately 10^7^–10^8^ colony-forming units per milliliter (CFU/mL).

### 2.3. Irradiation Device

The light source device used for photodynamic therapy was obtained from PineTek (PINETEK LLC, 511 University Dr., College Station, TX, USA) and assembled with support from the Technical Support laboratory at the Physics Institute of São Carlos (USP/SP/Brazil). The system consists of a plate holder equipped with twenty-four light-emitting diodes (LEDs) arranged in a 4 × 6 configuration, delivering an irradiance of 75 Mw/cm^2^ at a wavelength of 660 nm. The LEDs provide uniform illumination across the surface of the plates. The distance between the LED light guide and the sample surface was maintained at 80 mm. The device allows precise control of both the light intensity and exposure time to ensure accurate dose delivery. Thermal stability is maintained through active cooling with an integrated electric fan and passive dissipation via heatsinks. The variation in irradiance between wells was maintained within 10%. A control board enabled adjustment of the operating current to deliver irradiance of 15, 30, or 60 J/cm^2^.

### 2.4. In Vitro Photodynamic Treatment of Planktonic K. pneumoniae

The procedure used was adapted from our previous work as follows. Bacteria seeded on BHI were cultured aerobically overnight at 35 °C. The inoculum was prepared in PBS and adjusted to an absorption of 0.2–0.3 at 600 nm (OD 600, Agilent Cary 60 UV-Vis, Agilent Technologies Inc., Santa Clara, CA, USA), corresponding to approximately 10^7^–10^8^ colony-forming units per milliliter (CFU/mL), and was deposited into a 24-well plate. Varying concentrations of PDZ (from 25 to 200 µg/mL) plus MB (ranging from 5 to 20 µg/mL) were added, in the presence or absence of antibiotic CIP (0.005–4 µg/mL), or GEN (0.5–16 µg/mL), or CEF (0.5–16 µg/mL). The PSs and antibiotics were chosen based on published results from previous experiments performed in our laboratory. Samples were irradiated for 3 min 20 s or 6 min 40 s, corresponding to total light doses of 15 or 30 J/cm^2^ [total light dose (J/cm^2^) = fluence rate (W/cm^2^) × treatment time (s)]. The control samples were subjected to identical treatment, in the absence or presence of PSs, and were either kept in darkness or irradiated to evaluate the effect related to the conditions mentioned herein. During photodynamic therapy, the 24-well plates remained covered with a lid, and special care was taken not to disturb the plate. After completion of the PDT protocol, samples and controls were assessed in serial dilutions of each suspension and were cultured on MH agar plates and incubated overnight at 35 °C. Various treatment time-sequences combining PDT, antibiotics, and both PSs were investigated. The specific experimental procedures are described alongside the corresponding results. The effectiveness of aPDT was assessed by counting the number of CFU/mL and comparing the results with those for controls. All experiments were carried out at least three times.

### 2.5. Statistical Analysis

All experiments were performed in triplicate and repeated three times. The results are expressed as the mean and standard deviation. Differences between groups were compared by analysis of variance with statistical significance at ≤0.05.

## 3. Results

The results of this study are presented in two parts, for single- and dual-PS systems.

### 3.1. Effect of PDT with Individual Photosensitizers in Combination with Antibiotics Against K. pneumoniae

We investigated whether combining aPDT with CIP, GEN, or CEF produces a synergistic bacterial reduction and whether the treatment sequence (aPDT followed by antibiotics vs. antibiotics followed by aPDT) would influence the outcome. Three antibiotics were initially evaluated at multiple concentrations, CIP (1, 2, and 4 µg/mL), GEN (4, 8, and 16 µg/mL), and CEF (4, 8, and 16 µg/mL), following 100 min exposure before photodynamic treatment with either MB (10 µg/mL) or PDZ (200 µg/mL) for 20 min under a constant light dose of 30 J/cm^2^ (Supplementary Figure S1A, experimental plan). Bacterial survival (log CFU/mL) was assessed after each treatment sequence. Dark controls with maximal concentrations of MB (10 µg/mL) or PDZ (200 µg/mL) showed no significant variation in *K. pneumoniae* cell viability. An analysis of light exposure up to 60 J/cm^2^ alone without photosensitizer revealed no appreciable bactericidal effect on *K. pneumoniae*. As shown in Supplementary Figure S1, MB combined with any antibiotic resulted in a rapid and complete reduction in *K. pneumoniae*, achieving undetectable CFU even at the lowest antibiotic concentrations tested (1 µg/mL for CIP, 4 µg/mL for GEN and CEF) (Supplementary Figure S1). In contrast, PDZ-mediated PDT produced a more gradual reduction in bacterial viability and required higher antibiotic doses to reach comparable killing (up to 4 µg/mL CIP, 8 µg/mL GEN, and >16 µg/mL CEF) (Figure S1). Overall, MB consistently demonstrated superior bactericidal efficacy compared with PDZ across all antibiotic combinations (Figure S1).

To assess whether the treatment sequence influenced antimicrobial efficacy, the order of application was reversed so that *K. pneumoniae* was first incubated with MB (10 µg/mL) or PDZ (200 µg/mL) for 100 min, followed by 20 min antibiotic exposure under a total irradiation dose of 30 J/cm^2^ (Supplementary Figure S2A, experimental plan). As shown in Supplementary Figure S2B, MB in combination with CIP again produced near-complete or complete bacterial eradication (log CFU/mL ≈ 0) (Figure S2B). In contrast, GEN exhibited a sequence-dependent response. When PS was applied first, GEN displayed complete *K. pneumoniae* killing even at the lowest tested concentration, whereas the reverse sequence resulted in a dose-dependent decrease in viability, with MB outperforming PDZ but achieving complete eradication only at the highest GEN concentration (Figure S2C). For CEF, the general pattern was consistent with MB demonstrating rapid and comprehensive bacterial clearance irrespective of sequence, while PDZ resulted in only a partial reduction in viable *K. pneumoniae* cells (Figure S2D). Overall, MB outperformed PDZ across all antibiotic combinations and application sequences tested.

**Figure 1:**
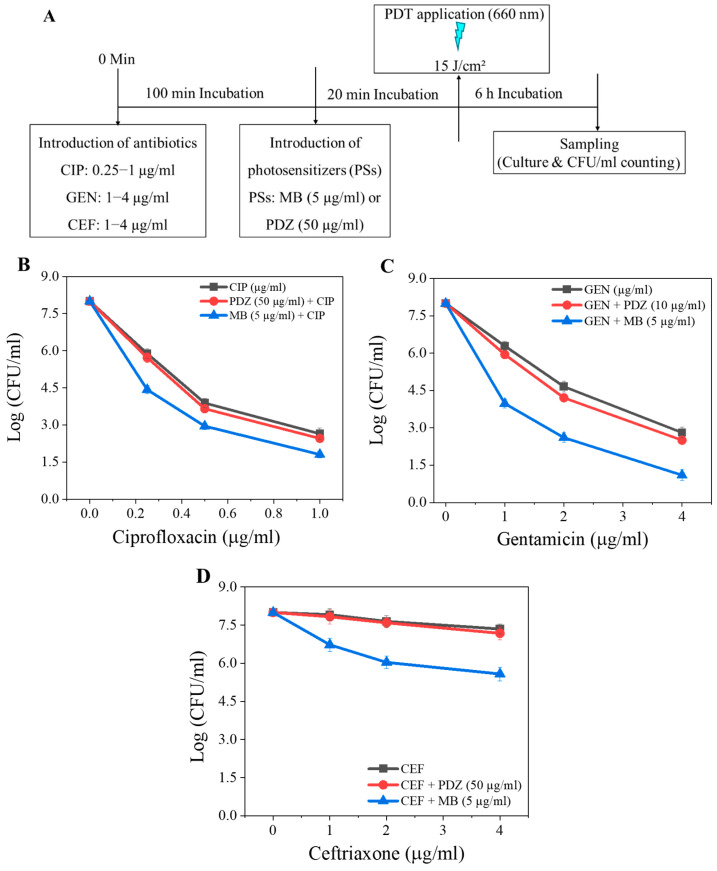
(**A**) The experimental workflow of antibiotic (CIP, GEN, and CEF) and individual photosensitizer (PS) treatments. The combined effects of CIP and PS (MB or PDZ) (**B**), GEN and PS (MB or PDZ) (**C**), and CEF and PS (MB or PDZ) (**D**) on the viability of *K. pneumoniae*. The graph shows colony-forming units (CFU/mL) as a function of antibiotic and PS concentrations (µg/mL) at an energy dose of 15 J/cm^2^. Statistical significance was determined by comparing treated groups (PS + antibiotics) to the “only antibiotic” control group for each concentration. Error bars represent the standard deviation across replicates.

Reducing both the antibiotic and PS doses, along with lower light influence (15 J/cm^2^), still resulted in marked enhancement of antimicrobial activity against *K. pneumoniae* (Figure 1A). CIP concentrations of 0.25–1 µg/mL demonstrated a clear dose-dependent reduction in viability (Figure 1B), with both PSs improving bacterial killing relative to CIP alone; MB remained consistently more effective than PDZ at all doses, achieving a 6.4 log_10_ reduction at 1 µg/mL (Figure 1B). A similar trend was observed for GEN, where increasing the GEN concentration (1–4 µg/mL) further reduced bacterial survival, and MB-mediated aPDT again outperformed PDZ, resulting in an approximately 7 log_10_ reduction at its maximal concentration of 4 µg/mL (Figure 1C). In contrast, CEF showed comparatively limited enhancement in combination with aPDT, with substantial viable counts remaining even at 4 µg/mL; however, MB still demonstrated greater efficacy (~2.4 log_10_ reduction) than PDZ (~0.8 log_10_ reduction) under equivalent conditions (Figure 1D). Overall, these findings confirm that even at reduced antibiotic and irradiation levels, aPDT significantly potentiates antibiotic activity, with MB providing the most pronounced bactericidal effect across all antibiotic combinations.

**Figure 2:**
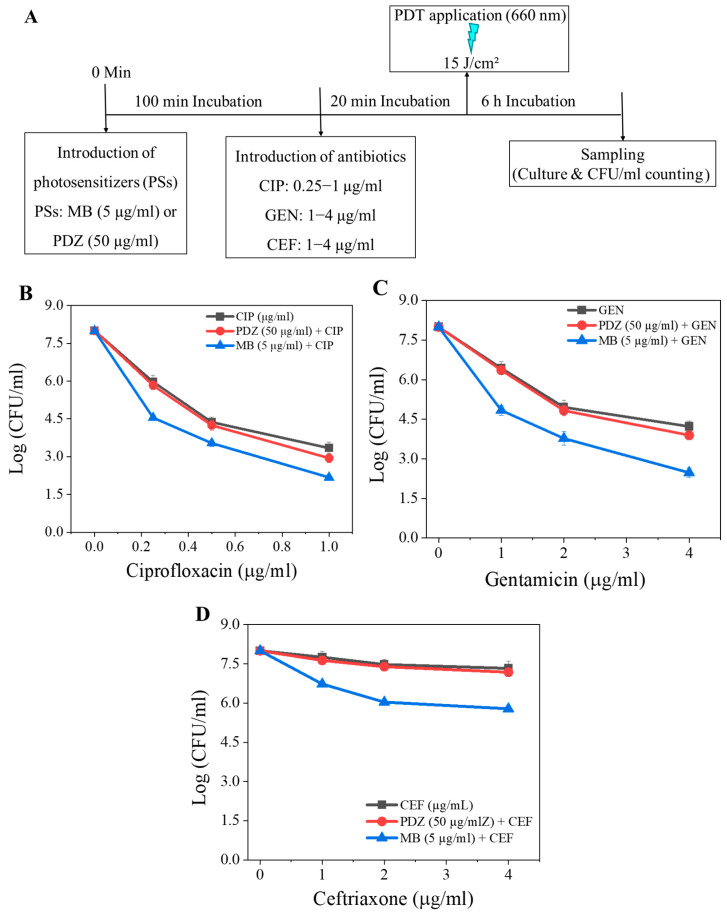
(**A**) The experimental workflow of individual photosensitizer (PS) and antibiotic (CIP, GEN, and CEF) treatments. The combined effects of PS (MB or PDZ) and CIP (**B**), PS (MB or PDZ) and GEN (**C**), and PS (MB or PDZ) and CEF (**D**) on the viability of *K. pneumoniae*. The graph shows colony-forming units (CFU/mL) as a function of PS and antibiotic concentrations (µg/mL) at an energy dose of 15 J/cm^2^. Statistical significance was determined by comparing treated groups (PS + antibiotics) to the “only antibiotic” control group for each concentration. Error bars represent the standard deviation across replicates.

To assess the impact of treatment order on antimicrobial performance, the application sequence was reversed, with *K. pneumoniae* first exposed to PS (MB, 5 µg/mL; or PDZ, 50 µg/mL) for 100 min before antibiotic treatment for 20 min, while maintaining a total light dose of 15 J/cm^2^ (Figure 2A). When MB preceded CIP exposure, the bacterial count was dose-dependently reduced by CIP, and a maximal reduction of 5.9 log_10_ was observed with a CIP concentration of 1 µg/mL (Figure 2B). Overall, this sequence resulted in reduced efficacy compared with the “antibiotic-first” (Figure 1B) configuration, possibly due to the antimicrobial pre-treatment weakening cellular defenses and enhancing susceptibility to oxidative damage. PDZ showed less potential compared to MB (Figure 2B). With GEN, the “PS-first” sequence also reduced bacterial viability, though slightly less effectively than the reverse order, particularly for PDZ at elevated GEN concentrations (Figure 1C vs. Figure 2C). For CEF, MB applied before the antibiotic treatment produced a more pronounced bactericidal effect across all doses compared with the “antibiotic-first” sequence, whereas PDZ showed minimal dependence on the treatment order, with comparable levels of CFU/mL reduction in both configurations (Figure 1D vs. Figure 2D).

### 3.2. Effect of PDT with Combined Photosensitizers in Combination with Antibiotics Against K. pneumoniae

**Figure 3:**
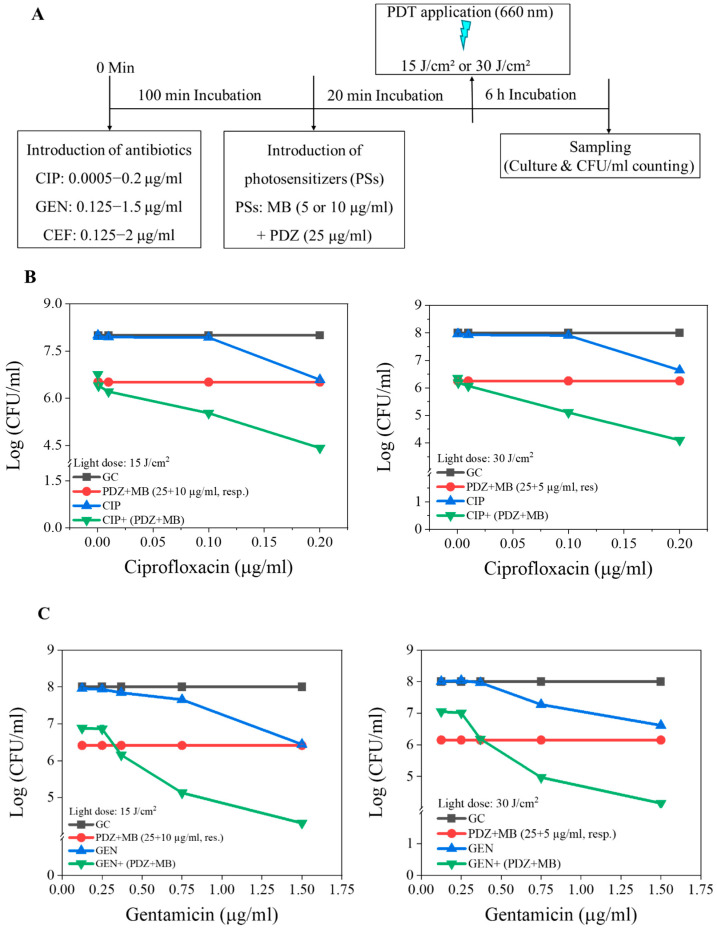

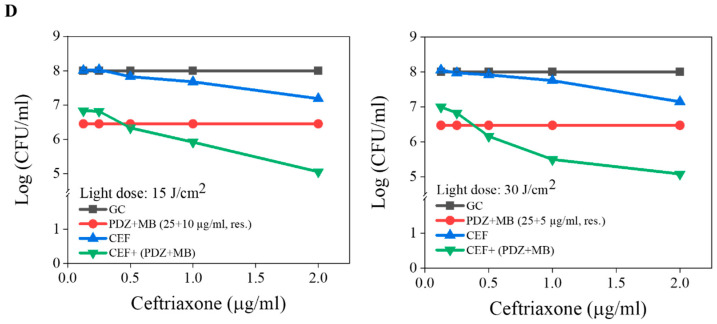
(**A**) The experimental workflow of antibiotic (CIP, GEN, and CEF) and dual-photosensitizer (PS) treatments. The combined effect of dual photosensitizers (MB plus PDZ) and the antibiotics CIP (**B**), GEN (**C**), and CEF (**D**) on the viability of *K. pneumoniae*. The graph shows colony-forming units (CFU/mL) as a function of dual-PS and antibiotic concentrations (µg/mL) at energy doses of 15 J/cm^2^ and 30 J/cm^2^. Statistical significance was determined by comparing treated groups (dual-PS + antibiotics) to the “only antibiotic” control group for each concentration. Error bars represent the standard deviation across replicates.

We next evaluated the antimicrobial efficacy of DaPDT combined with the antibiotics CIP, GEN, and CEF. Sub-inhibitory conditions were selected to maximize the potential detection of synergy. In these experiments, antibiotics were applied before PS exposure (Figure 3A), as this sequence had previously demonstrated superior performance compared with the reversed order. Dual-PS combinations of PDZ:MB at either 25:10 µg/mL or 25:5 µg/mL were used under irradiated light doses of 15 J/cm^2^ and 30 J/cm^2^, respectively. These concentrations were selected below their established maximal phototoxic thresholds to enable reliable evaluation of potential synergistic, additive, or antagonistic interaction. Because enhanced killing alone does not establish true synergy, interaction strength was quantified using the fractional inhibitory concentration index (FICI), calculated from the fractional doses of each agent in combination relative to their individual effective doses.

Figure 3B summarizes the effects of CIP combined with dual PSs on the viability of *K. pneumoniae* under both irradiation conditions. Each panel compares four treatment groups: general control (GC), PDZ + MB alone, CIP alone, and CIP with dual PSs (MB + PDZ) across CIP concentrations of 0–0.2 µg/mL (Figure 3B). Across both irradiation conditions, the combined treatment consistently produced a greater reduction in CFU counts, with efficacy increasing with the CIP concentration (Figure 3B). Individually, DaPDT and CIP exhibited only mild bactericidal activity after 18 h of incubation (Figure 3B). In contrast, combining DaPDT with relatively lower concentrations of CIP resulted in a synergistic effect (Figure 3B). At an irradiance of 15 J/cm^2^, CIP (0.2 µg/mL) alone produced a 1.42 log_10_ reduction, and PDZ:MB (25:10 µg/mL) yielded a 1.49 log_10_ reduction. Their combination resulted in a 3.60 log_10_ reduction, exceeding the expected additive effect (2.91 log_10_) by 0.69 log_10_, confirming synergism (Figure 3B). Similarly, at 30 J/cm^2^, CIP (0.2 µg/mL) reduced viability by 1.36 log_10_, and DaPDT (25:5 µg/mL) reduced it by 1.74 log_10_; the combined treatment produced a 3.90 log_10_ reduction, 0.80 log_10_ above the additive expectation (3.10 log_10_). In both cases, the combined regimen markedly outperformed monotherapy (Figure 3B). Further, at 0.2 µg/mL CIP, the synergy was most pronounced, especially at 30 J/cm^2^, where enhanced killing was achieved despite a lower MB dose (Figure 3B).

The GEN + DaPDT combination produced a potent dose-dependent reduction in *K. pneumoniae*, markedly exceeding the effects of either one alone (Figure 3C). PSs alone had a moderate impact, whereas increasing the light dose from 15 J/cm^2^ to 30 J/cm^2^ amplified bacterial killing. At 15 J/cm^2^, GEN (1.5 µg/mL) and PDZ:MB (25:10 µg/mL) reduced the bacterial counts by 1.56 and 1.59 log_10_, respectively, but their combination achieved a 3.69 log_10_ reduction, 0.54 log_10_ above the additive expectation (Figure 3C). Similarly, at 30 J/cm^2^, GEN (1.5 µg/mL) and PDZ:MB (25:5 µg/mL) reduced viability by 1.39 and 1.85 log_10_, respectively, while their combination reached 3.85 log_10_, 0.61 log_10_ above the additive expectation, confirming strong synergism (Figure 3C).

Combining CEF with DaPDT produced a potent reduction in *K. pneumoniae* viability at both light doses of 15 J/cm^2^ and 30 J/cm^2^ (Figure 3D). Across all CEF concentrations, the combination consistently outperformed either treatment alone, indicating synergism (Figure 3D). The response was dose-dependent, with no significant killing at the lowest concentration (0.25 µg/mL) of the antibiotic, but more than 2.96 log_10_ reduction at the highest concentration (1.5 µg/mL) using the DaPDT (Figure 3C). At 15 J/cm^2^, CEF (2 µg/mL) and PDZ:MB (25:10 µg/mL) reduced bacterial viability by 0.81 and 1.55 log_10_, respectively, whereas the combination achieved a 2.96 log_10_ reduction, 0.6 log_10_ above the additive expectation (Figure 3D). At 30 J/cm^2^, CEF (2 µg/mL) and PDZ:MB (25:5 µg/mL) produced reductions of 0.85 and 1.53 log_10_, while their combination reached a 2.91 log_10_, exceeding the additive value by 0.54 log_10_ (Figure 3D). Increasing the light dose from 15 J/cm^2^ to 30 J/cm^2^ further enhanced killing in the combination group despite halving (from 10 µg/mL to 5 µg/mL) the MB concentration, indicating the robustness of the synergistic interaction. All FICI values confirmed the synergism across all CEF + dual-PS combinations, with no evidence of antagonism. Together, these findings demonstrate that CEF and dual-PS aPDT act synergistically to enhance bacterial inactivation.

## 4. Discussion

The rise of multidrug-resistant (MDR) bacterial infections continues to undermine current therapeutic options, highlighting the urgent need for novel, mechanism-diverse strategies. Antimicrobial photodynamic therapy (aPDT) offers a promising adjunct to conventional antibiotics because it generates ROS through pathways distinct from drug-specific targets [16]. Such multimodal pressure diminishes the likelihood that pathogens will simultaneously evade all mechanisms of action. This rationale is particularly relevant for *Klebsiella pneumoniae*, a high-priority MDR Gram-negative pathogen and the focus of this study. Despite the broad susceptibility of Gram-positive bacteria to a wide range of PSs, Gram-negative species such as *K. pneumoniae* are considerably more refractory due to their complex, highly anionic outer membrane [13]. Effective inactivation often requires higher PS concentrations or intense irradiation, which may be impractical in clinical or environmental settings. To improve individual aPDT performance under feasible treatment conditions, this study employed MB and PDZ, two PSs with robust individual aPDT performance and complementary wavelength absorption (~650 nm) that enables efficient co-activation using a single 660 nm light source. Our previous findings demonstrated that the simultaneous application of the PSs MB and PDZ resulted in a dose-dependent reduction in MB’s activity against *K. pneumoniae* [11]. Sequential treatments revealed that applying PDZ prior to MB caused only a slight antagonistic effect compared to MB alone, whereas the reverse sequence nearly eliminated MB’s efficacy [11]. Our previous observations indicate that the interaction between PSs is complex and not inherently additive [11]. Despite the lack of synergy between the PSs themselves, their potential in combination with antibiotics remains highly significant and largely unexplored. Therefore, the present study investigated the potential advantages of combining dual PSs with antibiotics against *K. pneumoniae*. A dark toxicity evaluation was performed without irradiation and showed that neither MB nor PDZ exhibited bactericidal activity against *K. pneumoniae* at any tested concentration. Similarly, red LED irradiation alone did not reduce *K. pneumoniae* CFU counts at either 15 or 30 J/cm^2^.

A key finding of this study is that the effectiveness of sequential antibiotic–PDT treatment heavily depends on both the antibiotic class and the physicochemical properties of the photosensitizer (PS). Cationic MB consistently exhibited superior bactericidal activity against *K. pneumoniae* compared to anionic PDZ, likely due to MB’s enhanced interaction with the negatively charged outer membrane, greater cellular uptake, and higher ROS-generating efficiency. This membrane association may also disrupt the efflux system, leading to increased intracellular antibiotic accumulation [17]. It is consistent with the idea that visible light primarily excites PS molecules located within or near the cell envelope, making the membrane an immediate and vulnerable target of ROS attacks. These surface-focused photochemical events probably initiate early structural damage that enables subsequent oxidative injury [8]. However, the exact spatial distribution and dynamic behavior of MB in *K. pneumoniae* remain poorly understood. High-resolution imaging and localization studies will be crucial to clarify PS–cell integration and to confirm the proposed mechanism of membrane-centered photodamage. Antibiotic-specific responses were observed: MB combined with CIP or GEN resulted in significant reductions in viability, whereas CEF showed limited standalone efficacy but improved markedly when used in combination (Figure 1). These findings are consistent with prior reports demonstrating enhanced antibacterial effects when aPDT is combined with conventional antibiotics across various bacterial species [18,19].

**Figure 4:**
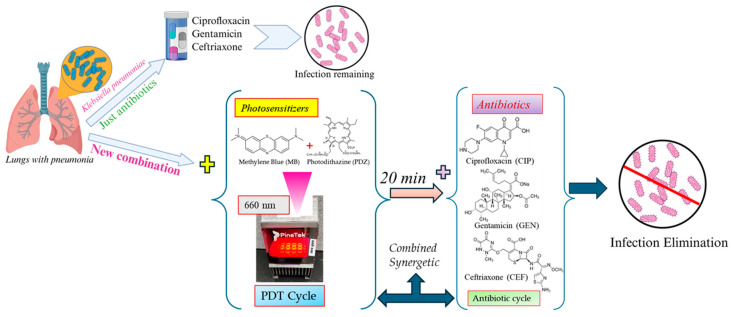
Schematic illustration of dual-photosensitizer antimicrobial photodynamic therapy (DaPDT) and its synergistic combination with antibiotics against *Klebsiella pneumoniae*.

The combination of CIP with DaPDT yielded clear synergistic interactions, particularly at the higher light dose (Figure 3B). CIP’s inhibition of DNA replication and the widespread oxidative damage induced by photoactivated PSs likely produce complementary disruption that exceeds their additive effect. Notably, sub-MIC CIP concentrations that were only modestly inhibitory (~1.5 log_10_ reduction) became highly effective when paired with dual PSs (~3 log_10_ reduction), underscoring the potential of DaPDT to restore antibiotic susceptibility while minimizing selective pressure for resistance. Increased ROS output at higher irradiance further amplified this synergy, demonstrating the importance of light dose optimization (Figure 3B). The combination of GEN with the DaPDT also produced potent bactericidal enhancement beyond their modality alone (Figure 3C). ROS-mediated membrane disruption likely facilitates aminoglycoside (GEN) uptake, while GEN’s inhibition of protein synthesis compromises bacterial recovery from oxidative injury. Interestingly, reducing the MB concentration from 10 to 5 µg/mL did not diminish the synergistic effect at 30 J/cm^2^, again highlighting the critical influence of irradiation parameters. Although aminoglycoside–PDT synergy has been noted in other pathogens [20,21,22], this study appears to be the first to report such interactions against *K. pneumoniae* using a dual-photosensitizer system. Cephalosporins exert their bactericidal activity by inhibiting peptidoglycan crosslinking, a mechanism fundamentally distinct from ROS-mediated damage [23]. Although CEF alone displayed limited activity against *K. pneumoniae*, its combination with DaPDT produced dose-dependent enhancement at both light doses, with a pronounced effect at 30 J/cm^2^ (Figure 3D). The synergy likely arises from oxidative injury that compromises membrane disruption and enhances β-lactam access to periplasmic targets, revealing a strategy for potentiating antibiotics otherwise constrained by poor uptake in Gram-negative bacteria [23]. The combined results of the present study are schematically presented in Figure 4.

Collectively, these findings demonstrate that DaPDT enhances the efficacy of CIP, GEN, and CEF against *K. pneumoniae*, with FICI values of ≤0.5, confirming synergistic interactions across antibiotic classes. The enhanced efficacy observed with the combined treatments may be attributed to the hypothesis that PSs act as substrates for bacterial efflux pumps. This competition between the DaPDT and antibiotics can reduce drug efflux and promote greater antibiotic uptake following membrane permeabilization [24]. The synergistic effect may also stem from cumulative ROS production, as both DaPDT and antibiotic exposure contribute to oxidative stress within bacterial cells [25]. Increasing the irradiation from 15 to 30 J/cm^2^ substantially reduced the required MB concentration, supporting the concept that controlled variation of light parameters can optimize the therapeutic window. From a mechanistic perspective MB and PDZ generate ROS via distinct mechanisms, with MB predominantly favoring Type I pathways (producing superoxide, hydrogen peroxide, and hydroxy radicals), while PDZ mainly operates through a Type II pathway yielding singlet oxygen (^1^O_2_) [26,27,28]. When used individually, MB’s photodynamic efficacy is strongly influenced by its cellular uptake and ability to generate mixed ROS, whereas PDZ relies primarily on high-efficiency ^1^O_2_ production that is dependent on localization and formulation [26]. In combination, MB and PDZ can produce complementary ROS profiles under the same irradiation wavelength of 660 nm, broadening oxidative stress through concurrent Type 1 and Type II mechanisms [26,27,28]. However, this dual-PS system may also be limited by photon competition, ROS quenching, and photobleaching effects that can partially reduce the net ROS yield [11,26]. When integrated with antibiotics, DaPDT-induced ROS can synergistically enhance membrane disruption and antibiotic uptake [28], resulting in an amplified oxidative burden and improved antibacterial efficacy against *K. pneumoniae*. A potential limitation is that unbound PS molecules in suspension may compete for excitation light, reducing the fraction of photons reaching cell-associated PSs, an effect that should be considered in dose modeling and device design [29]. Importantly, the bidirectional enhancement, lower antibiotic requirements, and reduced PS doses provide a compelling framework for minimizing toxicity, slowing resistance evolution, and improving therapeutic efficacy. By partially restoring susceptibility to clinically relevant antibiotics, DaPDT may reduce reliance on last-line agents and improve outcomes in MDR infections [30]. While this approach is ideally suited to localized or accessible infections, optical delivery barriers limit its use in deep tissues. For internal infections, alternative activation strategies such as chemiluminescent or sonodynamic excitation may extend the utility of this platform. To our knowledge, this is the first study to investigate the synergistic potential of combining antibiotics with DaPDT and to demonstrate enhanced killing of *K. pneumoniae*, highlighting a promising strategy for improving antimicrobial therapies against multidrug-resistant pathogens.

## 5. Conclusions

Overall, our study supports the feasibility and promise of integrating antimicrobial photodynamic therapy (aPDT) with conventional antibiotics to enhance bactericidal efficacy against *Klebsiella pneumoniae*. Combinations of methylene blue (MB)-mediated aPDT with fluoroquinolones or aminoglycosides produced the most pronounced synergistic effects, while the comparatively modest potentiation observed with cephalosporins such as ceftriaxone warrants further mechanistic investigation. A key outcome of this work is the demonstrated synergy between sequential antibiotic administration and MB- or PDZ-based aPDT activated by red light, resulting in significantly reduced antibiotic concentrations required to achieve bactericidal effects relative to antibiotic treatment alone.

Future studies should aim to elucidate the mechanistic basis of this synergy through the targeted assessment of reactive oxygen species generation, quantification of DNA and membrane damage, and evaluation of alterations in cell physiology following treatment. In addition, validation in more physiologically relevant and clinically challenging models, including multidrug-resistant strains, biofilms, and an in vivo respiratory infection system, will be critical for establishing the translational applicability of this therapeutic approach. To our knowledge, this is the first study to systematically evaluate multiple aPDT–antibiotic combinations against *K. pneumoniae*. Although limited to planktonic cultures and in vitro analyses, this study provides compelling support for continued exploration of aPDT–antibiotic regimens as a promising strategy to enhance therapeutic outcomes and potentially reduce the selective pressure that drives the emergence and persistence of antimicrobial resistance.

## Data Availability

The original contributions presented in this study are included in the article/Supplementary Material. Further inquiries can be directed to the corresponding authors.

## Supplementary Materials

The following supporting information can be downloaded at https://www.mdpi.com/article/10.3390/pharmaceutics18050587/s1, Figure S1: (**A**) Experimental workflow of antibiotics (CIP, GEN, and CEF) and individual photosensitizers (PSs) treatment. The combined effect of CIP and PSs (MB or PDZ) (**B**); GEN and PSs (MB or PDZ) (**C**), and CEF and PSs (MB or PDZ) (**D**), on the viability of *K. pneumoniae*. The graph shows colony-forming units (CFU/mL) as a function of antibiotic and PSs concentrations (μg/ml) at an energy dose of 30 J/cm^2^. Statistical significance was determined by comparing treated groups (PS + antibiotics) to the “only antibiotic” control group for each concentration. Error bars represent standard deviation across replicates; Figure S2: (**A**) Experimental workflow of individual photosensitizers (PSs) and antibiotics (CIP, GEN, and CEF) treatment. The combined effect of PSs (MB or PDZ) and CIP (**B**); PSs (MB or PDZ) and GEN (**C**), and PSs (MB or PDZ) and CEF (**D**), on the viability of *K. pneumoniae*. The graph shows colony-forming units (CFU/ml) as a function of PSs and antibiotic concentrations (μg/ml) at an energy dose of 30 J/cm^2^. Statistical significance was determined by comparing treated groups (PS + antibiotics) to the “only antibiotic” control group for each concentration. Error bars represent standard deviation across replicates.
