# Supplementary material for: Dual-Photosensitizer Antimicrobial Photodynamic Therapy (DaPDT) and Its Combination with Antibiotics: A New Investigation Modality Against Klebsiella pneumoniae
Source: Pharmaceutics. 2026 May 9;18(5):587. doi: 10.3390/pharmaceutics18050587 (PMC13210737; doi:10.3390/pharmaceutics18050587)
Supplement: Supplementary file 1 [file pharmaceutics-18-00587-s001.zip › pharmaceutics-4278308-supplementary.pdf]

Supplementary data: Dual-Photosensitizer Antimicrobial Photodynamic Therapy (DaPDT) and Its Combination with Antibiotics: A New Investigation Modality Against *Klebsiella pneumoniae*

Koteswara Rao Yerra[1,*] and Vanderlei S. Bagnato[1,2,*]

*Correspondence: ykrao@tamu.edu (KRY); bagnatovs@tamu.edu (VSB)

Figure S1.

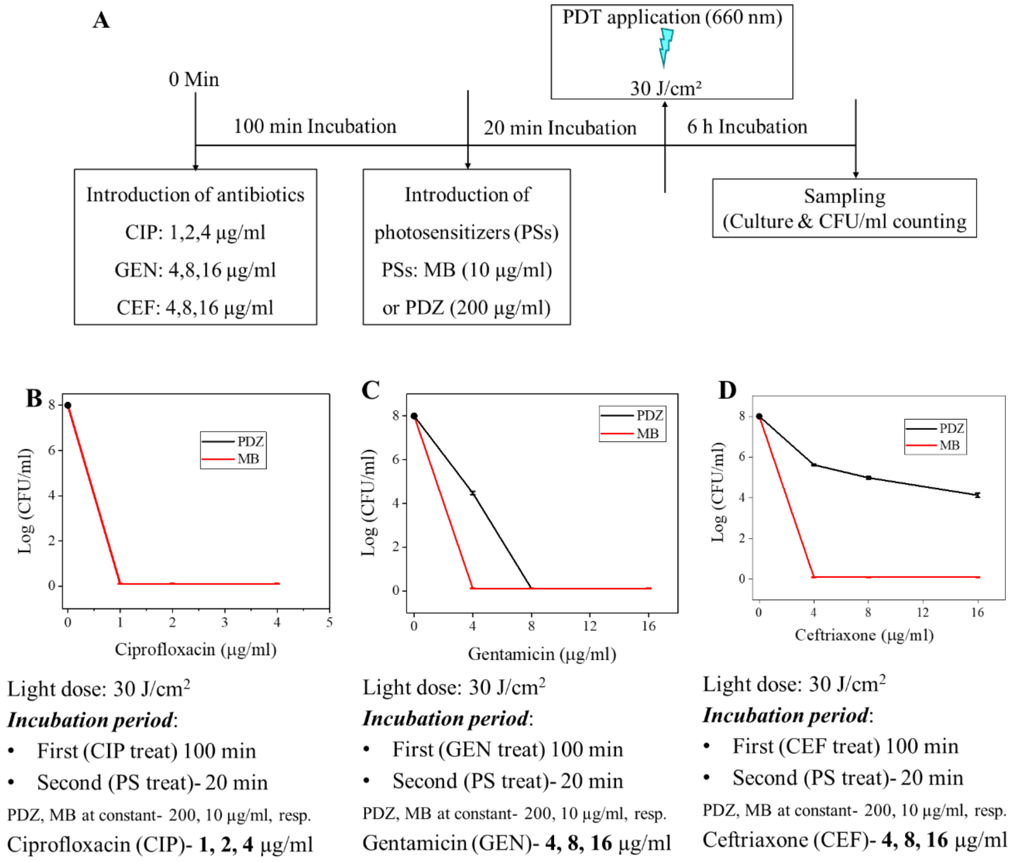

Figure S1. (**A**) Experimental workflow of antibiotics (CIP, GEN, and CEF) and individual photosensitizers (PSs) treatment. The combined effect of CIP and PSs (MB or PDZ) (**B**); GEN and PSs (MB or PDZ) (**C**), and CEF and PSs (MB or PDZ) (**D**), on the viability of *K. pneumoniae*. The graph shows colony-forming units (CFU/mL) as a function of antibiotic and PSs concentrations (µg/ml) at an energy dose of 30 J/cm². Statistical significance was determined by comparing treated groups (PS + antibiotics) to the "only antibiotic" control group for each concentration. Error bars represent standard deviation across replicates.

Figure S2.

A

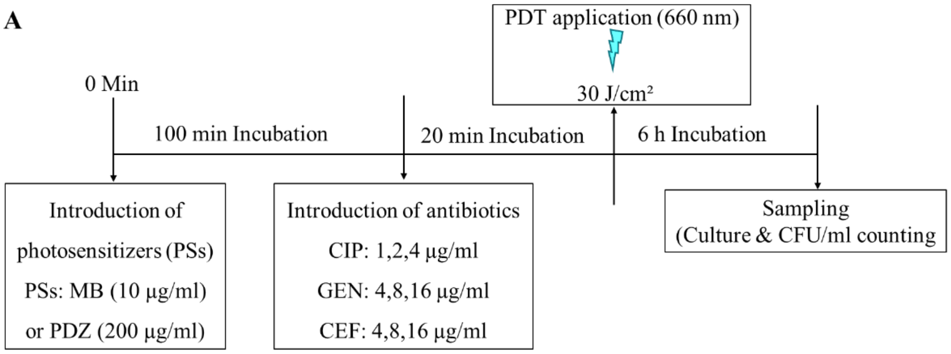

(B)

Antibiotic first:

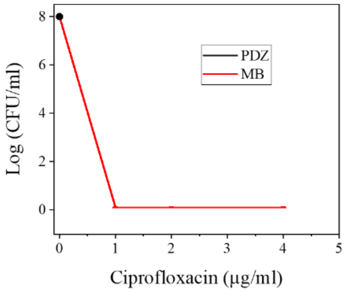

Light dose: 30 J/cm^2
Incubation period:
- First (CIP treat) 100 min
- Second (PS treat)- 20 min

PDZ, MB at constant- 200, 10 µg/ml, resp.
Ciprofloxacin (CIP)- **1, 2, 4** µg/ml

PS first:

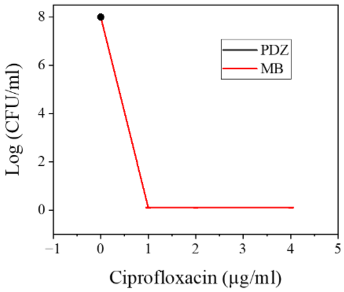

Light dose: 30 J/cm^2
Incubation period:
- First (PS Treatment)- 100 min
- Second (CIP)- 20 min

PDZ, MB at constant- 200, 10 µg/ml, resp.
Ciprofloxacin (CIP)- **1, 2, 4** µg/ml

(C)

Antibiotic first:

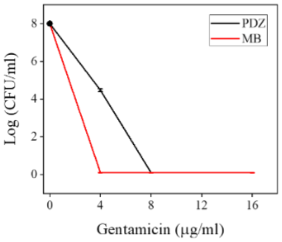

Light dose: 30 J/cm^2
Incubation period:
- First (GEN treat) 100 min
- Second (PS treat)- 20 min

PDZ, MB at constant- 200, 10 µg/ml, resp.
Gentamicin (GEN)- **4, 8, 16** µg/ml

PS first:

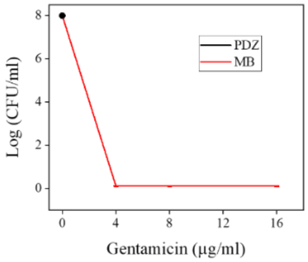

Light dose: 30 J/cm^2
Incubation period:
- First (PS Treatment)- 100 min
- Second (GEN)- 20 min

PDZ, MB at constant- 200, 10 µg/ml, resp.
Ciprofloxacin (CIP)- **4, 8, 16** µg/ml

(D)

Antibiotic first:

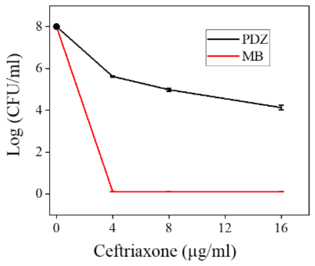

Light dose: 30 J/cm^2

Incubation period:

- First (CEF treat) 100 min
- Second (PS treat)- 20 min

PDZ, MB at constant- 200, 10 μg/ml, resp.

Ceftriaxone (CEF)- **4, 8, 16** μg/ml

PS first:

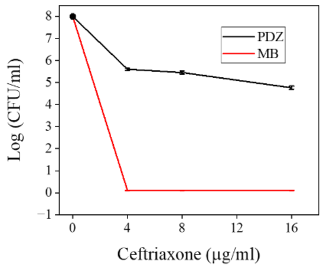

Light dose: 30 J/cm^2

Incubation period:

- First (PS Treatment)- 100 min
- Second (CEF)- 20 min

PDZ, MB at constant- 200, 10 μg/ml, resp.

Ciprofloxacin (CIP)- **4, 8, 16** μg/ml

Figure S2. (**A**) Experimental workflow of individual photosensitizers (PSs) and antibiotics (CIP, GEN, and CEF) treatment. The combined effect of PSs (MB or PDZ) and CIP (**B**); PSs (MB or PDZ) and GEN (**C**), and PSs (MB or PDZ) and CEF (**D**), on the viability of *K. pneumoniae*. The graph shows colony-forming units (CFU/ml) as a function of PSs and antibiotic concentrations (μg/ml) at an energy dose of 30 J/cm^2. Statistical significance was determined by comparing treated groups (PS + antibiotics) to the "only antibiotic" control group for each concentration. Error bars represent standard deviation across replicates.
